# Psychological Effects and Quality of Life in Parents and Children with Jia-Associated Uveitis

**DOI:** 10.3390/children9121864

**Published:** 2022-11-30

**Authors:** Silvana Guerriero, Roberta Palmieri, Francesco Craig, Francesco La Torre, Valeria Albano, Gianni Alessio, Massimo Corsalini, Paola Lecce, Andrea De Giacomo

**Affiliations:** 1 Department of Basic Medical Sciences, Neurosciences and Sense Organs, Institute of Ophthalmology, University of Bari, Piazza Giulio Cesare 11, 70124 Bari, Italy; 2 Department of Basic Medical Sciences, Neurosciences and Sense Organs, Institute of Child Neuropsychiatry, University of Bari, Piazza Giulio Cesare 11, 70124 Bari, Italy; 3 Department of Culture, Education and Society, University of Calabria, 87036 Cosenza, Italy; 4 Unit for Severe Disabilities in Developmental Age and Young Adults, Scientific Institute, IRCCS E. Medea, 72100 Brindisi, Italy; 5 Department of Pediatrics, Pediatric Rheumatology Center, “Giovanni XXIII”, Pediatric Hospital, Via Giovanni Amendola 207, 70126 Bari, Italy; 6 Interdisciplinary Department of Medicine (DIM)-Section of Dentistry, “Aldo Moro” University of Bari, 70124 Bari, Italy

**Keywords:** juvenile idiopathic arthritis, uveitis, quality of life, parent stress, coping, resilience

## Abstract

Juvenile idiopathic arthritis (JIA) is a chronic inflammatory disease common in children and young adults. Uveitis is the most frequent serious extra-articular JIA manifestation and can lead to severe ocular complications, vision loss, and permanent blindness. This study aims to evaluate the psychological condition and the quality of life of children affected by JIA associated with uveitis (JIA-U) and the repercussion of this condition on parents. Thirty children and adolescents with active uveitis (Uveitis group) and comorbid joint symptoms of JIA were referred to the Unit of Ophthalmology, Giovanni XXIII Hospital of Bari, and 30 age-matched healthy controls (Healthy group) were enrolled with their parents. Four questionnaires were administered: Child Behaviour Checklist (CBCL), Parent Stress Index in Short Form (PSI), Pediatric Quality of Life Inventory (PedsQL), and Coping Inventory for Stressful Situations (CISS). The data were collected from February 2021 to December 2021. No significant differences between the two groups in CBCL, PSI, or CISS tests were shown (*p* > 0.05). Conversely, significant differences between the two groups were observed in the PedsQL (*p* < 0.05). This study shows how several ocular complications, recurrent eye examinations, and the rigor of long-term treatment may negatively influence health-related quality of life in children with JIA-U.

## 1. Introduction

Juvenile idiopathic arthritis (JIA) is a chronic inflammatory disease defined as arthritis of up 6 weeks of duration without any specific cause that affects children and young adults under 16 years [1]. This pathology is characterized by an incidence of 8.2/100,000 for the population under 16 years old and by an annual prevalence of approximately 70.2/100,000 [2,3,4,5,6,7,8,9,10,11,12,13,14,15,16,17,18,19,20,21,22,23,24,25,26,27,28,29,30,31,32,33,34,35,36,37,38,39,40,41,42,43,44].

The supported etiology is an immunogenic mechanism with the involvement of genetic and environmental factors [5]. Uveitis is the most frequent serious extra-articular manifestation associated with JIA [6]. Although the eye is known as an immune-privileged organ, the breakdown of the blood–aqueous humor barrier induces non-infectious uvea inflammation [7]. Uveitis related to JIA (JIA-U) accounts for 47% of all types of uveitis in children [8], approximately 1/10,000 of the world population [9].

The most common forms of JIA associated with uveitis are the oligoarticular (four or fewer joints involved) and polyarticular (five or more joints affected) types. Specifically, the oligoarticular type develops uveitis at a higher rate (20%) with respect to the polyarticular (5%) kind [10].

According to the Standardization of Uveitis Nomenclature (SUN) Criteria, active uveitis features associated with JIA include anterior chamber cells, anterior chamber flare, vitreous cells, and haze [11]. Other clinical findings are band keratopathy, posterior synechiae, cataract, glaucoma, and macular edema [12].

To date, there are no international consensus statements specifically relating to the diagnosis and treatment of JIA-U [13].

Generally, the management depends on clinical practice, with an extensive variation in terms of investigation and therapeutic strategies, according to uveitis experts [14].

Importantly, JIA is strongly associated with psychosocial distress, especially when associated with uveitis [15,16,17]. In fact, the sudden or insidious symptom onset, trend of remission and recrudescence, visual loss, chronic therapy (topical, systemic, or biological molecules), and the short or medium–long-term eye complications of uveitis have a strong psychological impact on the quality of life of children and their families.

In this perspective, the significance of investigating psychological changes in patients with chronic diseases is well-acknowledged [18,19], as well as the parental stress in managing chronic physical illness in children.

Notably, although previous literature works have effectively demonstrated how chronic diseases (i.g., diabetes, cystic fibrosis, asthma, cancer, epilepsy, sickle cell disease) might lead to particular psychological changes, few studies investigated the psychological implications associated with uveitis [20,21,22,23].

Understanding the measure of parents’ stress, as well as strategies used as adaptations, can lead to improvements in the therapeutic and family support approach. The aim of this study is to achieve four endpoints: (1) evaluation of the quality of life of children and adolescents with JIA-U (Pediatric Quality of Life Inventory, PedsQL), (2) investigation of behavior in young patients with JIA associated with uveitis from parents’ point of view (Child Behaviour Checklist, CBCL, test), (3) the establishment of the parents’ stress rate (Parent Stress Index in Short Form, PSI-SF stairs), (4) the search of strategies applied by their family to cope with stress (Coping Inventory for Stressful Situations, CISS, scale) and comparing them with children without chronic diseases and their families. The research hypothesis of this study is that the JIA-U worsens the quality of life of children, impacting their emotional condition and inducing behavioral problems. Furthermore, it is supposed that the parents of these patients exhibit an increased stress level.

## 2. Methods

### 2.1. Study Design

This is a case–control study to compare children and adolescents with oligoarticular JIA-associated uveitis (Uveitis group), their parents, and healthy controls (Healthy groups). The psychometric comparison of multi-item questionnaires was assessed.

### 2.2. Participants and Setting

The Uveitis group comprised 32 children and adolescents with active uveitis and the comorbidity of JIA who were referred to the Unit of Ophthalmology, Giovanni XXIII Hospital of Bari, and their parents. Of these selected cases, 30 were included because of meeting the eligibility criteria, while 2 cases were dropped out. These 2 uveitis cases were ones with only cataracts and another one with cataracts and glaucoma.

The healthy group consisted of 30 children and adolescents that were randomly recruited, based on the availability of parents and subjects to participate in the study, from schools located in Bari and Foggia.

The study was conducted from February 2021 to December 2021. Each visit was conducted every 2 months during this period.

### 2.3. Ethical Considerations

The data were treated in accordance with the tenets of the Declaration of Helsinki. All parents signed informed consent. Ethical approval for the study was obtained from the Interregional Ethics Committee (n.6440, 22 January 2021).

### 2.4. Eligibility Criteria

The first criterion was for patients with oligoarticular JIA in accordance with the ILAR criteria [24]. Active uveitis at the ophthalmic visit during the enrolled period, including at least anterior chamber cells and/or anterior chamber flare and/or vitreous cells and/or haze and/or macular edema, according to the SUN criteria [11]. Patients with cataracts and/or glaucoma were ruled out because of exacerbated visual impairment.

Patients having no other systemic or ocular disease that could potentially affect vision, and being under the age of 18 years, were included in the study.

### 2.5. Data Collection Instruments

We administered validated questionnaires such as the Child Behaviour Checklist (CBCL) [25], the Parent stress Index in short form (PSI) [26], the Pediatric Quality of Life Inventory (PedsQL) [27], and the Coping Inventory for Stressful Situations (CISS) [28,29]. Parent and patient-based questionnaires were completed in person after the ophthalmic examinations at the clinic. A member of the research team was available to read the questions to subjects who requested them.

### 2.6. Assessment

The assessment was carried out by administering standardized scales, the “PSI”, the “CBCL”, the “PedsQL”, and the “CISS”:

The PSI Short Form (PSI/SF) is a reliable questionnaire designed to measure parental stress and difficulties with the parenting role. It is a summary form of the Parenting Stress Index (PSI) full-length test [26]. All 36 items on the Short Form are contained in the Long Form and are written at a 5th-grade reading level. Each item requires the parent to respond with a statement on a five-point Likert scale (1 = Strongly Agree, 2 = Agree, 3 = Not Sure, 4 = Disagree, and 5 = Strongly Disagree). The PSI consists of three subscales: the parenting distress (PD) scale, the dysfunctional interaction parent–child (P-CDI) scale, and the difficult child (DC) scale. The PD scales define the level of distress that a parent perceives in his parenting role. The P-CDI scale expresses the parent’s perception of a child who does not respond to his or her expectations and, therefore, of a non-gratifying interaction with the child. The DC scale values how much the parent perceives his child as easy/difficult to manage. The PSI-SF produces subscale raw scores ranging from 12 to 60 and an overall parenting stress total score that ranges from 36 to 180; a higher score indicates a greater level of stress. A score above the 90th percentile indicates a clinically significant level of parenting stress. The total stress (TS) scores, obtained by the sum of the scores of the 3 subscales, is an index of total parenting stress. The test also includes a Defensive Responding (DF) scale that indicates the parent tends to give a better self-image, minimizing the problems and the perceived stress in the relationship with the child.

The CBCL is a common questionnaire used to assess emotional and behavioral problems in children [25], as rated by parents, and includes CBCL/6–18 and CBCL/1 ½-5 for different ages.

The first section of the scale includes 20 items related to a child’s participation in sports, hobbies, games, activities, organizations, jobs, chores, friendships, social interactions during play, independent work, and school functioning. The second section consists of 120 items on behavior or emotional problems during the past 6 months. The main areas investigated are aggression, hyperactivity, bullying, conduct problems, defiance, and violence. Responses are recorded on a Likert scale: 0 = Not true, 1 = Somewhat or Sometimes true, 2 = very true or often true. Lower scores indicate lower functioning on the academic performance and adaptive functioning scales. Higher scores indicate higher levels of maladaptive behavior on the syndrome, total problems, externalizing, and internalizing scales.

The PedsQL is a validated measure of general health-related quality of life (QoL) in children and adolescents from 2 to 18 years of age [20]. It consists of four scales measured on a 5-point scale: 1: physical functioning (PF) 8 items, 2: emotional functioning (EF) 5 items, 3: social functioning (SF) 5 items; and 4: school functioning (ScF) 5 items. The questions consider how many problems occurred in children in the last month with a Score range from 0 to 100; higher scores indicate better QoL. Child self-report includes ages 5 to 7, 8, to 12, and 13 to 18 years. Parent self-report includes ages 2 to 4 (toddler), 5 to 7 (young child), 8 to 12 (child), and 13 to 18 (adolescent). A 5-point response scale is utilized: 0 (never a problem), 1 (almost never a problem), 2 (sometimes a problem), 3 (often a problem), and 4 (almost always a problem). To further increase the ease of use for the young child self-report (ages 5–7), the response scale is simplified to a 3-point scale: 0 (not at all a problem, 2 (sometimes a problem), 3 (a lot of a problem). The parent self-report includes toddler age range, which does not include the self-report form and only 3 items for the school functioning scale. Items are reverse-scored and transformed from 0 to 100 (0 = 100, 1 = 75,2 = 50, 3 = 25, 4 = 0). A higher score indicates a better Health related-QoL (HRQoL).

The CISS measures the following three types of coping styles: Task-Oriented Coping (dealing with the problem at hand), Emotion-Oriented Coping (focusing on consequent emotions), and Avoidance-Oriented Coping (Distraction and Social Diversion) [28,29]. The CISS includes adolescent and adult forms. They include 48 items and use a five-point response format. The adolescent version of the CISS is suitable for individuals between the ages of 13 and 18. The adult version of the CISS is suitable for individuals who are 18 years of age and older. For this study, we used only the adult version.

The disease activity of arthritis was calculated using the Juvenile Arthritis Disease Activity Score (JADAS) [24]. The JADAS includes the following items: physician’s global assessment of disease activity, measured on a 0–10 visual analog scale (VAS), where 0 is no activity, and 10 is maximum activity; parent/patient global assessment of child well-being, measured on a VAS 0–10, where 0 is very well, and 10 is very poor; the erythrocyte sedimentation rate (ESR), normalized to a 0 to 10 scale; and the count of joints with active disease developed in three versions depending by the total assessed: JADAS10, JADAS27, or JADAS71 [30]. Because oligoarticular JIA has fewer than 10 joints involved, JADAS10 was chosen and routinely performed. The JADAS10 is based on the count of any involved joint, up to a maximum of 10 joints [31]. The JADAS10 is calculated as the simple summation of its four items, which can give a total score of 0–40 [30,31]. However, the feasibility of these instruments is enhanced by the presence of the cutoffs for identifying high and low levels of activity [32,33]. They were developed for oligoarticular and polyarticular JIA separately. The cutoffs were first developed for all the original JADAS versions, and they considered the remission (inactive) disease, low (minimal), moderate, and high disease activity [32,33].

At each visit, the uveitis findings were evaluated according to the SUN Criteria [11]. Furthermore, the visual acuity was evaluated as best-corrected visual acuity (BCVA), measured by charts at a distance of 4 m and calculated in the logarithm of the minimum angle of resolution (LogMar).

### 2.7. Statistical Analysis

Descriptive statistics were used to summarize the variables studied and the characteristics of the subjects. The differences among demographic variables were evaluated by the chi-squared test (sex). Non-parametric tests (Mann–Whitney) were used to examine the differences in age, PSI/SF, CBCL, CISS, and PedsQL between the groups. A *p*-value of less than 0.05 was considered statistically significant. For statistical processing, we used the data processing program the Statistical Package for Social Science, Version 20.0.

## 3. Results

The socio-demographic characteristics of the Uveitis-associated JIA group and the Healthy control group are summarized in Table 1.

The analysis of the emotional and behavioral problems of the patients included in the study did not show significant statistical differences between the two groups, as shown in Table 2.

The mean number of ophthalmic visits was 5 (range 3–6), each one every 2 months. In the Uveitis group, a notable visual impairment was recorded. The mean BCVA in the Uveitis group was 0.35 LogMar. No considerable variations were noted in uveitis findings related to visual impairment.

In all of the JIA patients, the JADAS10 was calculated. In each visit, the JADAS10 was in remission disease activity (0–1) for all of the patients, and the articular activity state was well controlled.

No statistically significant differences were found in the variables analyzed in the PSI and CISS questionnaire, as shown in Table 3 and Table 4.

The analysis conducted to evaluate differences in QoL highlights statistically significant differences in all variables of the PedsQL questionnaire. In particular, significant data were found in the variables of PF (*p* = 0.007), EF (*p* < 0.0001), SF (*p* = 0.032), and ScF (*p* < 0.0001). They are summarized in Table 5.

**Figure 1:**
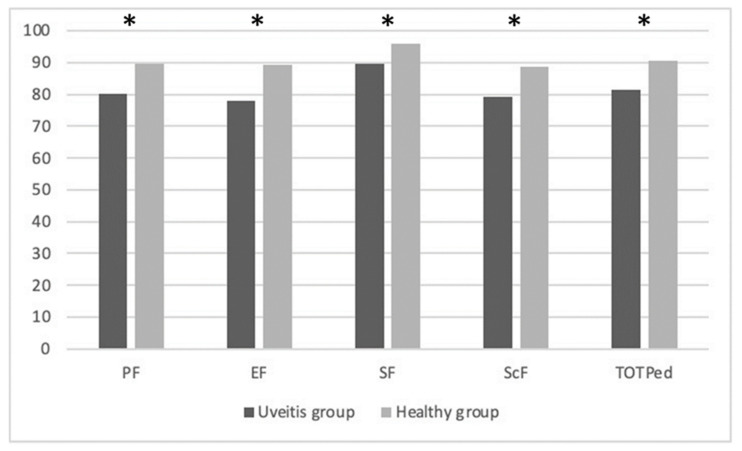
Explained the PedsQL mean scores differences between groups. * significant data.

Figure 1 below explains the PedsQL mean scores differences between the groups: physical functioning (PF), emotional functioning (EF), social functioning (SF), school functioning (ScF), and Total Scale Score (TOTPed).

## 4. Discussion

JIA-U is a pathology that frequently appears as asymptomatic or is not perceived by the children, even if it is in an advanced stage. Therefore, visual loss or ocular complications have to be often managed during the initial phase of the clinical treatment, sometimes recurring to surgery [34].

In fact, in order to monitor the progression of the pathology from its initial stage, the American Academy of Pediatrics Section on Rheumatology and Section on Ophthalmology recommended ophthalmology screenings every 3–4 months until 7 years of age to monitor the disease development, considering that in childhood there is a higher risk of developing uveitis for the youngest with JIA [35].

A small number of studies examined the impact of QoL in patients with JIA-U [16,17,34,36]. Particularly, most studies concerned solely adult patients [15,37,38], whereas very few studies have investigated children, teens, and their parents.

This study shows how several ocular complications, recurrent eye examinations, and the rigor of long-term treatment may negatively influence health-related quality of life in children with JIA-U. In particular, the considerable visual impairment associated with several ocular conditions and the frequency of ophthalmology visits may also affect the results of the CBCL, PSI, CISS, and PedsQL. In all of the JIA patients, the JADAS10 was in remission disease activity (0–1). Therefore, the results of CBCL, PSI, CISS, and PedsQL seem to be affected only by uveitis.

Notably, to the best of the authors’ knowledge, the present work is the first to compare JIA-U patients and their parents with a healthy control group.

Analyzing the responses to the PedsQL questionnaire, significant results emerged in all domains: PF, EF, SF, and ScF. These results highlight that uveitis has a major impact on the quality of life and affects every aspect of a child’s life. These findings are in agreement with previous studies [17,39,40,41,42,43].

In particular, a study reviewed groups of children (and their parents) with visual impairment due to ocular conditions except uveitis, showing that comments from 510 (44%) of 1163 children and 1078 (55%) of 1952 parents related to the QoL, such as psychosocial, impact on the school learning (in particular in reading skills), expectations and frustrations, dependency, and participation [39]. Another study conducted a semi-structured interview on children aged 6 to 12 and their parents; the impact on school, social factors, and emotional reactions were investigated, considering clinical evidence and therapeutic strategies practiced [40].

A recent study had proven a worsening of overall parameters of the QoL among children with a visual impairment, as measured by the PedsQL, version 4.0.6 [41].

These studies show that uveitis is indeed associated with worse physical and mental health-related QoL in children because of additional important medical stressors in this population [17,42]. The management of uveitis consists of complicated examinations, which can be frightening for children, and complex regimens of topical and systemic medications, which can be difficult to follow. Parents, in fact, report that children have difficulty understanding treatment regimens. Furthermore, children with uveitis may need to miss numerous school days for eye treatments and often may miss school for long periods because of complications. Many children report difficulty in compensating for missed course work, and this obviously has a negative impact on their academic performance. Absences from school also result in fewer opportunities to socialize with peers and the loss of friends. These children spend less time with their peers due to the high frequency of eye examinations and treatments, leading to reduced relationality. In addition, vision impairment may affect the ability to play some sports and to take part in play and leisure activities [39].

Uveitis and general chronic illness in childhood can also lead to the slower development of autonomy, close relationships with parents, and high levels of parental involvement. These children are vulnerable to being unable to manage their disease enough to ask for the help of parents even in adulthood. For all these reasons, children with uveitis experience negative emotions such as sadness, anxiety, anger, and resignation for the future, according to the results of our study.

It is worth highlighting that the attention and care of the family are fundamental for the correct adherence to the therapy [42].

Chronic disease had a strong impact on the child’s parents. A study showed a greater total stress score for mothers of children with JIA as measured by the PSI (235.4; 95% CI 218.5–252.3) than the mean total stress scores for mothers of healthy children (222.8; 95% CI 221.4–224.2) [44].

On the contrary, no significant results emerged in parenting distress by the PSI. The main stress source for parents was to compound work with family routine. It is worth highlighting that the measures have been carried out during the COVID-19 pandemic; thus, many parents worked from home and had to face logistical problems due to the closure of schools, as the management of their children in the context of personal autonomy, the preparation of meals, the support for school activities, and the oversight of free time. In addition, further causes of stress were, in many cases, related to the loss of work and to a direct experience with COVID-19. Hence, it could be supposed that the SARS-CoV-2 pandemic may have impacted parental stress, explaining why the results show high parenting stress but comparable between the two investigated groups (i.e., Uveitis and healthy groups).

The statistical analysis of the CBCL questionnaire did not show significant results. It appears from our data that chronic disease such as JIA-U does not cause emotional and behavioral problems in children. To the best of the authors’ knowledge, there are no other studies that focused on CBCL in children with JIA-U. Coping with anxiety was reported instead in adolescents [45].

The importance of screening for psychological changes in even younger children derived from the possibility that chronic disease might lead to anxiety and depression in adulthood [46,47,48,49]. For this reason, it is indeed important to evaluate the psychological health of these children through frequent screening.

To the authors’ knowledge, this study is also the first that studied the adaptation of the parents to the child’s clinical conditions (CISS Test). In this perspective, parents’ adaptative skills may be crucial to manage the clinical condition since even the child’s behavior depends on it. However, no significant results emerged from the CISS. Several studies show instead that the use of positive coping strategies is considered a necessary step in achieving resiliency and successful adaptation to stress [50,51].

Agreeing with a previous study, these results suggest that parents help their children when they focus coping efforts on altering controllable factors [41]. In fact, various examples of resilience were detected in all children and parents of the Uveitis group enrolled.

Some limitations of our study should be noted. The sample size is limited, and only one parent for each child was interviewed (more mothers than fathers). Finally, the interviews were performed during the first and second waves of the COVID-19 pandemic; thus, it could be possible that the restrictions associated with the pandemic may have affected the stress levels of the general population and acted as a confounding factor in the collected data. Hence, further studies should be performed to enlarge the sample size and to avoid external confounding factors in order to confirm the generalization of the findings of this study.

## 5. Conclusions

Children with JIA-U have decreased quality of life and visual functioning, which worsens with severe disease. Several ocular complications, eye examinations, and the rigor of long-term treatment influence health-related quality of life in these children. Our findings support the need for a screening-related quality of life and the further evaluation of the long-term psychological health in the JIA-U patients and the establishment of interdisciplinary collaboration, including psychological counseling.

## Figures and Tables

**Table 1:** Social demographic characteristics of patients and their parents of the two groups.

	Uveitis Group	Healthy Group	*p* Value
Number	32 (2 *)	30	
Gender			0.606
M	13	14	
F	17	16	
Mean age, years (SD)	9.77 ± 3.57	9.33 ± 4.498	
Mean BCVA (LogMar)	0.30–0.40	0–0.1	
Mean ophthalmic visits (N)	5 (range 3–6)		
Mother (N)	26	27	
Father (N)	4	3	

* drop out.

**Table 2:** Differences scores between groups found in CBCL.

	Uveitis Group	Healthy Group	Mann-Whitney U	Z	*p* Value
age	9.77 ± 3.57	9.33 ± 4.50	403,500	−0.690	0.490
Anxious/depressed	56.73 ± 9.95	53.43 ± 6.28	382,500	−1.210	0.226
Withdrawn	52.87 ± 5.32	52.20 ± 5.12	409,000	−0.798	0.425
Somatic complaints	55.10 ± 9.59	52.47 ± 5.46	407,500	−0.777	0.437
Attention problems	51.17 ± 4.09	53.37 ± 7.23	388,500	−1.401	0.161
Aggressive behavior	51.73 ± 4.29	52.23 ± 5.20	435,000	−0.329	0.742
Affective problems	53.50 ± 7.71	53.97 ± 7.38	427,500	−0.449	0.654
Anxiety problems	55.00 ± 9.54	53.60 ± 6.93	446,500	−0.068	0.946
ADHD	51.67± 5.14	53.17 ± 6.70	405,000	−0.986	0.324
Oppositional Defiant Problems	51.54 ± 4.26	53.60 ± 7.23	411,000	−0.826	0.409
Internalizing problems	55.37 ± 9.93	52.90 ± 5.83	417,000	−0.642	0.521
Externalizing Problems	52.00 ± 4.28	52.13 ± 4.78	447,000	−0.064	0.949
Total problems	54.07 ± 8.37	52.90 ± 5.58	449,000	−0.019	0.984

**Table 3:** Differences scores between groups found in PSI.

	Uveitis Group	Healthy Group	Mann-Whitney U	Z	*p* Value
Parenting distress	24.90 ± 7.43	27.50 ± 10.17	393,500	−0.837	0.403
Dysfunctional interaction parent–child	21.50 ± 6.28	19.97 ± 6.89	373,000	−1.141	0.254
Difficult child	24.77 ± 8.07	23.70 ± 7.88	413,500	−0.540	0.589
Defensive responding	15.93 ± 5.61	17.50 ± 6.31	379,000	−1.053	0.292
Total Stress	70.93 ± 20.53	70.30 ± 19.47	441,500	−0.126	0.900

**Table 4:** Differences scores between groups found in CISS.

	Uveitis Group	Healthy Group	Mann-Whitney U	Z	*p* Value
Task	48.03 ± 10.80	46.90 ± 10.04	406,000	−0.651	0.515
Emotion	49.07 ± 11.74	47.47 ± 12.18	432,500	−0.259	0.796
Avoidance	49.37 ± 8.86	52.83 ± 10.11	362,500	−1.295	0.195
Distraction	50.80 ± 9.01	50.60 ± 10.04	433,000	−0.252	0.801
Social diversion	46.80 ± 8.77	50.03 ± 11.86	355,500	−1.401	0.161

**Table 5:** Differences scores between groups in PedsQL.

	Uveitis Group	Healthy Group	Mann-Whitney U	Z	^†^*p* Value
Physical functioning ^†^	80.17 ± 14.86	89.53 ± 12.57	269,500	−2.703	0.007
Emotional functioning ^†^	77.83 ± 12.98	89.33 ± 16.07	207,000	−3.635	0.000
Social functioning ^†^	89.67 ± 14.08	96.00 ± 12.55	285,500	−2.858	0.004
School functioning ^†^	79.37 ± 17.36	88.67 ± 17.90	311,500	−2.151	0.032
TOTped ^†^	81.55 ± 9.97	90.60 ± 11.38	204,000	−3.509	0.000

^†^*p* < 0.005.

## Data Availability

The data presented in this study are available on request from the corresponding author.
